# Enhanced Elderberry Snack Bars: A Sensory, Nutritional, and Rheological Evaluation

**DOI:** 10.3390/foods12193544

**Published:** 2023-09-23

**Authors:** Ioana Mariana Haș, Dan-Cristian Vodnar, Alexa Florina Bungau, Alexandra Georgiana Tarce, Delia Mirela Tit, Bernadette-Emőke Teleky

**Affiliations:** 1 Doctoral School of Biomedical Sciences, University of Oradea, 410087 Oradea, Romania; ioanahas@gmail.com (I.M.H.); pradaalexaflorina@gmail.com (A.F.B.); 2 Institute of Life Sciences, University of Agricultural Sciences and Veterinary Medicine, 400372 Cluj-Napoca, Romania; bernadette.teleky@usamvcluj.ro; 3 Department of Food Science and Technology, University of Agricultural Sciences and Veterinary Medicine, 400372 Cluj-Napoca, Romania; 4 Medicine Program of Study, Faculty of Medicine and Pharmacy, University of Oradea, 410073 Oradea, Romania; tarce_alexandra@yahoo.com; 5 Department of Pharmacy, Faculty of Medicine and Pharmacy, University of Oradea, 410028 Oradea, Romania

**Keywords:** hedonic, freeze-dried elderberry, flow and deformation behaviour, sensory evaluation, functional snack bars

## Abstract

Interest in functional foods is continuously increasing, having the potential to be an ally in reducing cardiometabolic risk factors. This study focuses on developing and evaluating oat- and millet-based snack bars enriched with freeze-dried elderberry powder (FDEBP), aiming to combine great taste with enhanced nutritional value, antioxidant properties, and prebiotic potential. The research encompassed a sensory evaluation, nutritional assessment, and rheological analysis of the snack bars. A hedonic test was conducted to gauge consumer preferences and overall liking, providing insights into taste, texture, and acceptance. Sensory evaluation revealed positive feedback from participants, and acceptance rating scores ranged from 7 to 8.04, the best score recorded by one of the enhanced bars with 1% FDEBP. The rheological analysis determined the bars’ dynamic storage modulus (G′) and loss modulus (G″), assessing the material’s elasticity and mechanical properties. Results showed that the incorporation of 0.5% and 1% FDEBP in the oat and millet snack bars significantly impacted their rheological properties, enhancing structural strength. Nutritional analysis demonstrated that the snack bars provided a complete mix of macronutrients required in a daily diet. The study sheds light on the potential of functional snack bars enriched with FDEBP, offering a delectable way to access essential nutrients and bioactive compounds in a minimally processed form, without the addition of sweeteners or additives, friendly to the gut microbiota.

## 1. Introduction

According to the World Health Organization (WHO), cardiovascular disease (CVD) continues to be the leading cause of death worldwide alongside diabetes, with a combined toll of 19.9 million lives claimed annually. Numerous epidemiological studies have identified various bioactive compounds as promising candidates for the prevention and management of cardiometabolic diseases [1,2].

Bioactive substances have been well known and explored since antiquity for their numerous nutritional and healing properties [3]. Recently, there has been a growing interest in food supplements and functional foods containing bioactive plant-based compounds [4,5,6]. Increased disease prevalence and lifestyle changes have driven high demand for functional foods enriched with health-beneficial components [7] such as polyphenols, antioxidants, bioactive peptides, probiotics, vitamins, and minerals to address conditions such as cardiovascular issues, diabetes, obesity, depression, gastrointestinal disorders, and malnutrition [8,9].

However, there is a great need for snacks fortified with bioactive compounds that are healthy and valuable from a nutritional point of view. Many snack bar options currently on the food market are intensively processed and full of sweeteners, food additives, and saturated fat [10]. It is necessary that beyond the supply of bioactive compounds, the functional product should be minimally processed, natural, with a clean label, produced concerning nature, and friendly with gut microbiota. Because only in this way the value of bioactive compounds is beneficial.

Polyphenols are secondary metabolites of plants, and we find them in food sources such as vegetables, fruits, nuts, cereals, spices and herbs, dark chocolate, tea, and wine [11]. They are primarily recognized for their antioxidant effect, but the benefits they bring do not stop there. They have proven their anti-inflammatory and antithrombotic activity over time, their involvement in glucose and insulin homeostasis, and in improving the lipid profile and endothelial function [12]. Much more recently, we have data on the prebiotic potential of phenolic compounds, an extremely important aspect, taking into account that gut microbiota have an essential role in human health [11,13].

EB is a rich source of phenolic compounds, but it is relatively understudied compared to other berries. However, the stability and absorption of these compounds during gastrointestinal digestion are essential factors in determining their therapeutic effects [14]. Our previous study found that EB contains various phenolic compounds, which have proven their antioxidant and antibacterial activity and a significant bioaccessibility (75%). Additionally, the study revealed the prebiotic potential of these compounds on certain bacterial strains, providing valuable insights for further research on the health benefits of black EB [15]. 

Very recent research showed that in the case of the need to supplement the intake of polyphenols, consumers would choose a functional food rather than a nutraceutical in a significantly high proportion (77.7%) [16].

The production of functional snack bars enriched with EB can offer several important health-related benefits, such as high antioxidant content, prebiotic potential, nutrient density, flavour, and variety. Antioxidants play a crucial role in neutralizing harmful free radicals in the body, potentially reducing the risk of chronic diseases such as heart disease and cancer [17]. EB have a distinct, pleasant flavour that can add a unique and enjoyable taste to snack bars. This variety can attract consumers looking for new and exciting flavour options, potentially increasing product appeal. In summary, producing functional snack bars enriched with EB aligns with consumer preferences for healthier, more natural snack options. 

The hedonic test is a crucial tool in sensory evaluation, enabling the assessment of consumer liking and acceptability of a food product. It provides valuable insights into consumer preferences, guiding product development and marketing strategies based on overall consumer satisfaction [18]. Consumer acceptability of foods is frequently assessed using the 9-point hedonic scale developed by Peryam and Pilgrim in 1957 [19]. The term “overall liking” refers to a holistic hedonic response, where consumers evaluate the appeal of sensory modalities such as colour, odour, taste, and texture.

Building upon our previous study [15], as well as the need for healthy and quality snack bars, we sought to explore the incorporation of FDEBP into oat and millet-based snacks. Additionally, we conducted a hedonic test to evaluate the overall sensory appeal and consumer preferences of these new snack formulations. By incorporating the FDEBP, we aimed to enhance the nutritional value and potentially introduce novel flavour profiles to the oat and millet snacks. The hedonic test allowed us to gather valuable feedback from participants on the taste, texture, and overall likability of the EB-infused snacks, helping us determine their market potential and consumer acceptance. This study represents a continuation of our efforts to develop innovative and nutritious snack options that cater to consumer preferences while capitalizing on the health benefits of EB. A snack bar with prebiotic and cardiometabolic protective potential, fortified with EB in a form that supports the stability of phenolic compounds is the result of minimal processing, contains no added sweeteners, and lacks additives. To our knowledge, this represents the first snack bar product fortified with EB that possesses all these attributes.

## 2. Materials and Methods

### 2.1. Materials

In this study, we examined snack bars enriched with EB, specifically black *Sambucus nigra* L. fructus, which is commonly known as EB. These EB are sourced from the natural flora of Romania’s Bihor County (coordinates 46°43′36.5″ N, 21°54′32.4″ E) and identified at the Pharmaceutical Botany Department of the Faculty of Medicine and Pharmacy, Oradea University, Romania. Harvested in September 2022, the EB were immediately frozen at −20 °C, followed by lyophilization using a Telstar Lyo Quest 55 plus lyophilizer (Azbil Group, Terrassa, Spain) at a temperature of −55 °C and a pressure of 0.001 mbar for 72 h. After lyophilization, the fruits were ground, sieved into a fine powder, and stored in the dark until before use [15]. This lyophilization process was chosen to ensure that the compounds remained resistant to oxidation and chemical changes. It offers the advantage of occurring at low temperatures and in the absence of air [20].

The base in which we incorporated the FDEBP was carefully designed to contain all three macronutrients required daily in the human diet. To achieve this, we used oat flakes (Crownfield, Lidl, Eisenach, Germany), with a composition of 13.5% protein, 58.7% carbohydrates, 7% fat, and 10% fibres, along with millet flakes (Solaris Plant, Bucharest, Romania) containing 13% protein, 80% carbohydrates, 3.4% fat, and 2.7% fibres. These are two gluten-free cereals suitable for individuals with gluten intolerance or allergies. For nuts and seeds, we included walnuts (Orlando’s Import-Export 2001, Bucharest, Romania) with 19.8% protein, 3.7% carbohydrates, 60% fat, and 6.6% fibres; peanut butter (Solaris Plant, Romania) with 29.6% protein, 11.6% carbohydrates, 46% fat, and 8.5% fibres; and shelled hemp seeds (Canah International, Salonta, Romania) with 34% protein, 3.1% carbohydrates, 48% fat, and 6% fibres. To add sweetness, we used plum magiun (Sonimpex Topoloveni, Topoloveni, Romania) with 1.9% protein, 58% carbohydrates, 0.5% fat, and 5.5% fibres or dates (Orlando’s Import-Export 2001, Romania) with 1.8% protein, 70% carbohydrates, 0.1% fat, and 7.3% fibres. All ingredients were purchased from hypermarkets.

### 2.2. Snack Formulations

Three different bases were formulated, either used alone or combined with 0.5% or 1% FDEBP. A total of nine formulations were evaluated for the production of functional snacks. 

The first base contained oat flakes, walnuts, shelled hemp seeds, and plum magiun. The second base was formulated with millet flakes, peanut butter, shelled hemp seeds, and dates, and the third was similar to the first base, replacing the grain source with millet. The formulation structure is shown in Table 1.

The ingredients were weighed according to the percentages found in Table 1 and then crushed with the help of a food processor (Philips, Amsterdam, The Netherlands). The exception was the millet flakes, which were added both in the second and third version of the bar in their intact form and manually, slowly incorporated into the mixture obtained from the rest of the ingredients. The realised batch size was 400 g for each of the 9 formulations. The snack bars were prepared in laboratory conditions in compliance with hygiene standards.

### 2.3. Rheological Analyses

Dynamic rheological measurements of the protein bars (oat and millet) enriched with FDEBP were carried out using an Anton Paar MCR 72 rheometer (Anton Paar, Graz, Austria) equipped with a Peltier plate–plate system (P-PTD 200/Air) and a temperature controller (set at T = 25 °C and 35 °C). The rheometer was equipped with a 50 mm diameter smooth parallel plate geometry (PP-50-67300). Initially, approximately 3 g of the sample was placed at the centre of the lower plate of the Peltier system, with a 1.5 mm gap between the plates, and allowed to rest for 5 min [21,22].

After the sample was supplied, any excess was removed, and silicon oil was added to prevent drying. Subsequently, oscillatory frequency sweep tests were conducted, covering angular frequencies ranging from 0.628 to 628 rad/s. These tests were performed to determine the dynamic storage modulus (G′, Pa) and loss modulus (G″, Pa) of the protein bars. The values of G′ and G″ provide insight into the material’s ability to store elastic deformation energy and the viscous portion of the material, respectively.

The study involved the determination of key rheological parameters, specifically the G′, GG″, and loss tangent (tanδ, calculated as the ratio of GG″ to G′). These parameters were quantified to provide insights into the material’s viscoelastic behaviour, indicating its ability to store and dissipate energy.

### 2.4. Sensory Evaluation

A total of 81 subjects, potential consumers, aged between 19 and 75 years (39.5% men; mean age 36.1 ± 13.7, and 60.5% women; mean age 32.1 ± 13.8) from both urban and rural areas were asked to evaluate a total of 9 samples according with 9-point hedonic scale, regarding appearance, colour, smell, texture, flavour, and sweetness [23].

Each subject was minimally trained before evaluation with an explanation about the usage of the hedonic scale (1: dislike extremely, 2: dislike very much, 3: dislike moderately, 4: dislike slightly, 5: neither like nor dislike, 6: like slightly, 7: like moderately, 8: like very much, 9: like extremely) and about the followed sensory attributes. Then, they were served ~4 g of each sample, coded with a code consisting of a letter and a number. Samples were served one at a time. Also, they were invited to drink water between samples to clean their mouths.

This study was conducted with the approval of the Ethics Committee of the Faculty of Medicine and Pharmacy of Oradea, University of Oradea, No. 24/February 2021.

### 2.5. Statistical Analyses

Each measurement was conducted in triplicate, and the results are presented as the mean value (±SD, *n* = 3). Statistical analysis was carried out using Graph Prism Version 8.0.1 (GraphPad Software Inc., San Diego, CA, USA) via a one-way ANOVA test with Tukey’s multiple comparisons test. Significance was determined at a 5% confidence level for differences in means. 

## 3. Results and Discussions

### 3.1. Nutritional Assessment

Starting from the significant importance of decreasing the cardiometabolic risk, preventing and treating cardiometabolic diseases, the benefits of polyphenols in this area, and also the consumer’s preferences, we formulated three different snack bar bases, either used alone or enriched with 0.5% or 1% FDEBP. Freeze drying or lyophilization removes moisture from frozen substances, creating products with low water content for extended storage while preserving sensory qualities and nutrient integrity [24]. Its advantages include the retention of heat-sensitive components, easy rehydration, and suitability for instant products such as snack bars due to stable structures and excellent solubility.

All the ingredients used were natural and processed at a minimum. 

Nutritional values were calculated using Eat&Track (Cluj-Napoca, România), a reliable Romanian nutrition software for professionals, in which we the data for all the products used in the formulation of the bars available. Values per 100 g product can be found in Table 2.

Protein content was found to range between 13.1 g/100 g and 13.8 g/100 g, carbohydrate content ranged between 35.7 g/100 g and 48.6 g/100 g, fat content ranged between 17.6 g/100 g and 29.8 g/100 g, and fibre content ranged between 5.4 g/100 g and 6.9 g/100 g. Regarding the addition of phenolic compounds via fortification with FDEBP, we recall the results of the high-performance liquid chromatography (HPLC-DAD-ESI-MS) analysis from our previous study. A total content of polyphenols of 41.28 mg/g of FDEBP was revealed (17.25 mg anthocyanins, 10.51 mg flavonols, 7.69 mg hydroxycinnamic acid derivatives, and 5.83 mg hydroxybenzoic acid derivatives) [13].

The consumption of snack bars has grown a lot in recent years, the main reason seeming to be the need for comfort and the ease with which they can be consumed. They are easy to handle and store. But even if the main essential aspects in choosing such bars are those related to appearance and taste, the consumer should also be directed towards their quality, more precisely on the content of nutrients and bioactive compounds [25].

Regarding bioactive compounds, the benefits of polyphenols on human health have been intensively studied in recent years, with a correlation between the consumption of foods rich in polyphenols and the decrease in cardiometabolic risk being emphasized [16]. One of our recent studies highlighted the content of phenolic compounds of EB from the spontaneous flora of Romania, with a significant total content of polyphenols of 41.28 mg/g of FDEBP and a 75% bioaccessibility index of contained polyphenols. The high antioxidant potential was revealed following the determinations made by four assay methods (DPPH, ABTS, FRAP, and CUPRAC); the highest value was obtained with the FRAP assay (185 ± 0.18 μmol Fe^2+^/g DW). Another important aspect followed in the study was the antimicrobial activity, evaluated on seven strains containing Gram-negative and Gram-positive bacteria and yeasts. The most sensitive to the antimicrobial activity of FDEBP were proven to be Staphylococcus aureus and Pseudomonas aeruginosa, with the lowest minimum inhibitory concentration (1.95 mg/mL). Additionally, the prebiotic potential of FDEBP was determined on *L. plantarum*, *L. casei*, *L. rhamnosus*, *L. fermentum*, and *S. boulardii*, with results showing a positive influence on cell growth of all five probiotic strains used. The most significant increase (*p* < 0.05) was recorded on *L. casei*, with a growth index of 152.44% for 1.5% FDEBP, very closely followed by 0.5% and 1% concentrations [15]. In another study, this time a longitudinal intervention conducted on 30 healthy human volunteers, an essential change in microbial diversity was noticed immediately after administering 600 mg/day of purified extract from elderberries. The relative abundance of *Akkermansia* spp. increased, in the case of some of the subjects, even after supplementation was stopped [26].

Regarding the content the content of nutrients, according to previous research carried out in the direction of snack bar foods that claim to offer health support, values of protein content between 4.3 g/100 g and 21.9 g/100 g, carbohydrates between 38.4 g/100 g and 74 g/100 g, and fats between 3.1 g/100 g and 22.2 g/100 g were identified [27,28]. The products developed in the current study have values within the limits of those shown earlier, with the exception of fats where the values are between 17.6 g/100 g and 29.8 g/100 g. This choice is based on the evidence supporting the consumption of nuts for cardiometabolic protective purposes. Several studies have highlighted a positive relationship between the consumption of one serving (28 g) of nuts daily, and the decrease in the risk of CVD by 21% and mortality from any cause by 22% [29]. In another study, a reduction of up to 27% in the risk of diabetes was revealed in people who consumed at least 5 portions of nuts per week [28].

The content of fibres in our snack-bars is between 5.4 g/100 g and 6.9 g/100 g; values found in the specific range of other snack bars are between 1 and 20.8% [27,28]. Numerous studies highlight that fibres play a significant role in preventing cardiovascular disease, diabetes, and obesity [30].

Scientific evidence associates a higher consumption of whole grains with a lower risk of cardiovascular diseases, high blood pressure, and type 2 diabetes, as well as obesity; replacing the consumption of refined grains, both fashionable these days, with whole grains can be an effective step in reducing the risk of cardiometabolic diseases [31].

We decided to use two different cereals, oat and millet, for reasons related to their respective nutritional composition, due to the different textures. In our perception, millet flakes are slightly crunchy and more “spectacular” than oatmeal flakes. Millet is an important food source whose production has continuously increased in recent decades. Widely consumed in Africa and Asia, it has increasingly gained the attention of Western countries because it does not contain gluten, thus being beneficial for people with celiac disease or gluten sensitivity [32]. Moreover, millet is a valuable source of all essential nutrients and fibre. It has a high protein content, more essential amino acids than most cereals, and is a source of complex carbohydrates, fats, vitamins, minerals (contains more calcium than any other cereal), and bioactive compounds [33]. The consumption of millet has been correlated with multiple health benefits, supporting the prevention of cardiometabolic diseases via antioxidant activity and reducing blood glucose and cholesterol values [34].

Oat, another whole grain, is grown mainly in Europe and North America. It is an excellent source of quality proteins, complex carbohydrates, and fibres, especially beta-glucan, a soluble fibre that can reduce cholesterol levels and normalize glycaemic values. Oats are also an excellent source of minerals, vitamins, and antioxidants. Magnesium, manganese, iron, selenium, zinc, phosphorus, and thiamine are some of the vitamins and minerals contained in oats. Regarding antioxidants, oats contain avenanthramides, a family of antioxidants found only in oats, which have proven their ability to reduce blood pressure [35]. Oats do not contain gluten, but they have avenin, a similar type of protein. However, studies claim that consuming moderate or even large amounts of oats can be well tolerated by most celiac disease patients [36,37]. Scientific evidence correlates the consumption of oats with the improvement in cardiometabolic risk markers, as well as the composition of the intestinal microbiota [38,39].

The consumption of nuts and seeds is associated with a decrease in the risk of cardiovascular diseases, diabetes, and metabolic diseases [40,41]. Walnuts are rich in nutrients; they contain 65% fat, are rich in omega-3 fats [42], approximately 15% protein, and have a low carbohydrate content, mostly fibre. Moreover, they are a good source of antioxidants and vitamins and minerals such as vitamin E, B6, folic acid, copper, phosphorus, and manganese, compounds known to protect against heart disease, cancer, and cognitive decline [43,44]. However, these antioxidant properties must be well balanced so that their effects are beneficial to the body [45].

Although peanuts belong to the legume family, their nutritional profile resembles tree nuts [29]. They are an excellent source of all macronutrients, providing vegetable protein (about 25%), carbohydrates, and healthy fats, with a good fibre intake. They are rich in vitamin E, B3, B6, folic acid, manganese, magnesium, copper, and antioxidants [46]. Peanuts, despite being a prevalent allergen, are a highly nutritious and cost-effective legume, rich in proteins (comprising 70% high-quality proteins), including important bioactive compound resveratrol, which positively influences inflammation, cardiovascular health, type 2 diabetes, and cancer prevention. Despite their high caloric content, peanuts, with their combination of oil, fibre, and protein can aid in weight control by providing sustained satiety, especially when roasted, which also enhances flavour and shelf life while producing aromatic compounds [29].

Hemp seeds are an excellent source of healthy fats and essential fatty acids. They are also an excellent source of protein, with about 25% of the energy value coming from protein, a significantly higher content than in the case of other seeds. The proteins are rich in all essential amino acids and are easy to digest. In addition, they provide vitamins and minerals such as vitamin E, magnesium, calcium, phosphorus, sodium, potassium, iron, and zinc, along with bioactive compounds [47]. 

We emphasize that in the formulation of the bars, only natural sources of sugars were used, more precisely, plums “magiun” and dates, seeking to create a product as friendly as possible to the intestinal microbiota.

Generally, the conventional snack bars sold in stores are typically made with sugar, glucose syrups, honey, or artificial sweeteners. It is well known that excessive sugar consumption is a significant risk factor for developing cardiovascular, metabolic, and neurological diseases and certain types of cancer [48]. The Western diet is already rich in added sugars and saturated fats and poor in vegetables, fruits, and unprocessed fibres, aspects that have been shown to affect the integrity of the intestinal microbiota, causing intestinal dysbiosis. And even if we are talking about caloric or non-caloric sweeteners, more and more studies highlight the negative impact of their consumption on the intestinal microbial ecosystem [39,49].

Dates are fruits with a low glycaemic index, a source of carbohydrates, fibres, proteins, minerals (calcium, selenium, magnesium, iron, potassium, phosphorus, and zinc), vitamin B complexes, and antioxidants [50]. The consumption of dates holds significant importance due to their potential positive health effects, including their rich nutrient content and potential role in supporting digestive health and providing antioxidants [51].

Magiun of Topoloveni is a Romanian traditional product, a kind of plum jam made from qualitative and fully ripe plums with no added sugars, sweeteners, or preservatives. For preparation, plums are boiled in special double-walled vessels, indirectly heated, and continuously mixed with wooden paddles. They are rich in carbohydrates, soluble and insoluble fibres, vitamins, especially vitamin C, and antioxidants [52]. The consumption of plum magiun is of great importance due to its potential health benefits, such as its high fibre and antioxidant content, which may contribute to improved digestion and overall well being [53].

It is important to also mention here the aspect of antinutrients, plant compounds that are the subject of several studies, based on the fact that they reduce the body’s ability to absorb nutrients. Phytates, lectins, tannins, lectins, and oxalates are the most important antinutrients found in plant-based food. According to scientific evidence, antinutrients become a concern only in the case of people whose eating style involves only uncooked plant foods [54].

### 3.2. Rheological Analyses

**Figure 1:**
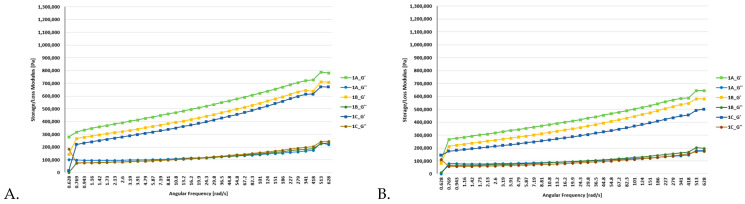
Flow and deformation behavior of the A1, A2, and A3 bars ((**A**). 25 °C and (**B**). 35 °C), where 1A—Oat, 1B—Oat + 0.5%EB, 1C—Oat + 1%EB.

**Figure 2:**
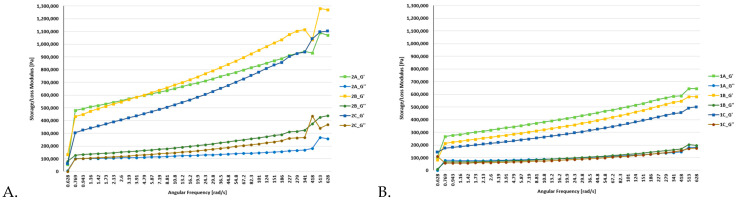
Flow and deformation behavior of the B1, B2, and B3 bars ((**A**). 25 °C and (**B**). 35 °C), where 2A—Millet, 2B—Millet + 0.5%EB, 2C—Millet + 1%EB.

**Figure 3:**
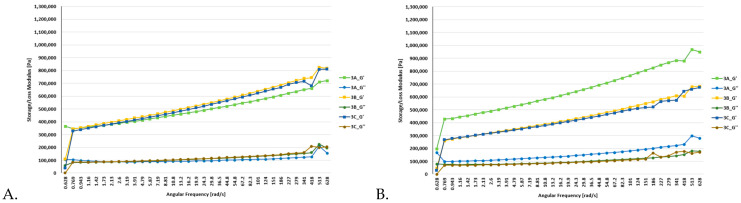
Flow and deformation behavior of C1, C2, and C3 bars ((**A**). 25 °C and (**B**). 35 °C), where 3A—Millet2, 3B—Millet2 + 0.5%EB, 3C—Millet2 + 1%EB.

Dynamic rheological measurements were performed on all batches of functional bars, which included the control bars (oat and millet) as well as the enriched bars (0.5% and 1% of FDEBP). These measurements were conducted both at 25 °C and at 35 °C (based on room and transportation temperatures of bars), as illustrated in Figure 1, Figure 2 and Figure 3. In the course of the frequency sweep, it becomes evident that as the angular frequency (ω) rises, there is a concurrent increase in the values of G′ and GG″.

Oat starch, characterized by unique properties such as small granule size, well-developed granule surface, high lipid content, and varying amylose and amylopectin proportions, is gaining attention for its diverse applications in both food and non-food industries, with its pasting and rheological properties playing a pivotal role in controlling product quality [55].

In all batches, G′ consistently exceeded G”, indicating a prevalent solid-like behaviour in the snacks [56]. These findings align with similar studies focusing on the production of functional foods [57]. The moduli were the lowest in the case of the oat-based snack bars, where the highest values were under 0.8 MPa at 25 °C and under 0.7 MPa at 35 °C.

In the process of crafting functional foods, it is crucial to manage the viscoelastic characteristics of the substrate [58]. Striking a balance is vital: excessive elasticity might hinder the snack bar lamination, while inadequate viscosity could lead to unsatisfactory spreading and physicochemical characteristics of the final snack bars. To comprehend the snack bar’s structural and textural attributes, conducting rheological tests employing both low-strain oscillatory shear conditions (mechanical spectra) and a wide stress range (stress sweeps) proves valuable [22]. 

Supplementary Tables S1–S6 display the viscoelastic properties of snack bars influenced by the addition of FDEBP, including variations in G′, GG″, and loss tangent (tanδ). Across all dough samples, G′ consistently surpassed GG″, signifying solid-like behaviour. Overall, plum magiun in samples 3A–3C and oat in samples 1A–1C led to a significant reduction in G′ and GG″ values while, notably, the addition of FDEBP increased the tanδ of all three types of snack bar mixtures. These findings imply that the addition of FDEBP resulted in a snack bar that was less elastic and more viscous compared to the control without FDEBP, decreasing the crosslinks within the gluten network and weakening the structure of the snack bars, which also provided a crunchy feeling. These effects can be attributed to the interchange among intermolecular disulfide bonds and modifications in molecular conformation [59].

The physicochemical characteristics of millet-based foods are shaped by the presence of accessible reactive groups and the exposure of their hydrophobic regions in a specific environment. The physicochemical interactions among food matrix components play a central role in the formation of gels, foams, and emulsions, affecting sensory attributes, shear resistance, and material flow during processing. Assessing the functional properties of millet-based foods in terms of these interactions can accelerate the development of high-quality functional food products and nutraceuticals, which can be further improved via deliberate chemical and enzymatic modifications, altering characteristics such as hydrophobicity, size, charge density, or the surrounding environment [60].

The addition of 0.5% and 1% of FDEBP had a noticeable impact on the dynamic rheological properties of the functional bars. The enriched snacks exhibited higher moduli, significantly surpassing the batch without any enrichment. 

In stress sweep experiments, the G′ values typically remain constant at low stress levels, forming a plateau region. Within this linear viscoelastic (LVE) range, G′ serves as a measure of the material’s elasticity under minimal deformation and represents its structural strength or mechanical rigidity at rest [56]. Only sample 2A with FDEBP showed some structural deformities, which were only observed at 35 °C.

### 3.3. Sensory Evaluation

**Figure 4:**
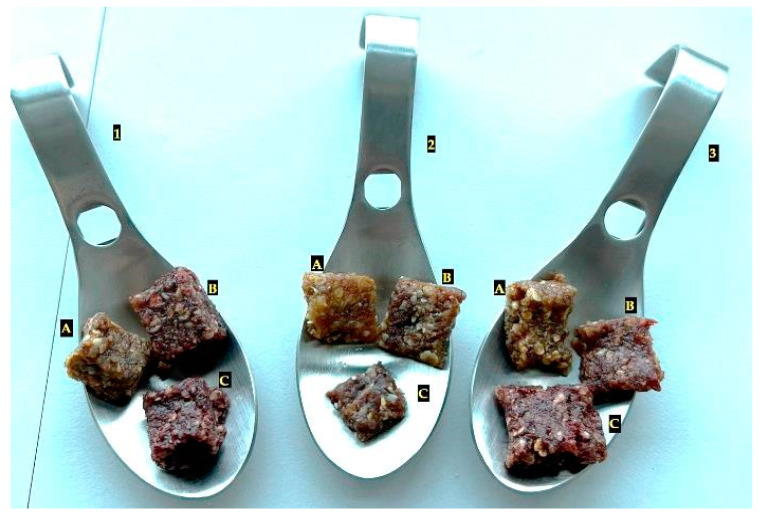
Oat- and millet-based FDEBP-enriched snack bars (where 1A—Oat, 1B—Oat + 0.5%EB, 1C—Oat + 1%EB; 2A—Millet, 2B—Millet + 0.5%EB, 2C—Millet + 1%EB, where 3A—Millet2, 3B—Millet2 + 0.5%EB, 3C—Millet2 + 1%EB).

Sensory characterization is essential as it enables evaluators to detect and describe the assessed products’ quantitative and qualitative attributes [61]. According to statistics, 74.4% of companies that intend to develop products with health-supporting characteristics and that emphasize selecting safe raw materials apply sensory evaluation tests. Researchers in the field claim that a group of 25–75 people is generally representative of assessing product acceptance in a new product’s development and optimization phases [62]. In the present study, the bars were evaluated for a group of 81 people, potential consumers. The snack bars are illustrated in the Figure 4.

**Figure 5:**
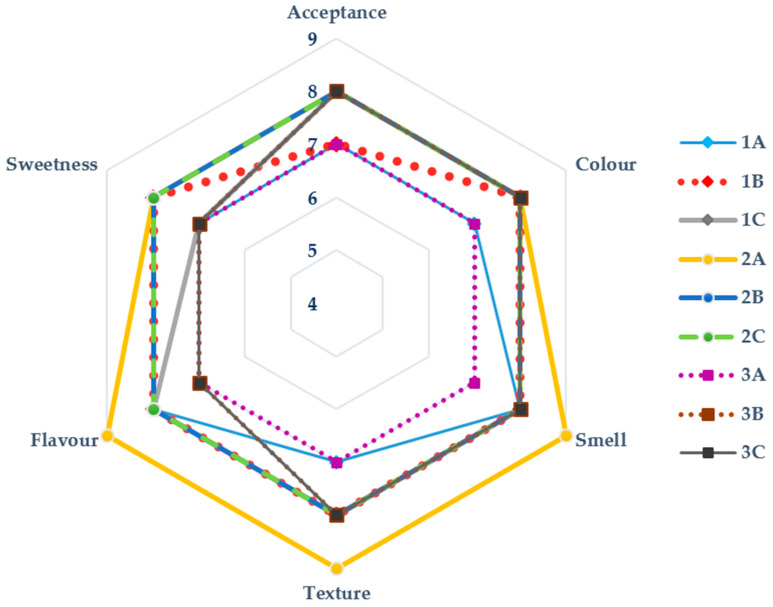
Effect of FDEBP in descriptive sensory evaluation of snack bars with various formulations (4: dislike slightly, 5: neither like nor dislike, 6: like slightly, 7: like moderately, 8: like very much, 9: like extremely).

The sensory evaluation of the snack bars (Figure 5) showed that those enriched with FDEBP obtained evaluation scores ranging from moderate to very good (6.96–8.19). It should also be emphasized that the control bars obtained, in most cases, lower scores than at least one of the variants improved with FBEBP, except for 2A, for taste, texture, smell, and sweetness.

The overall sensory acceptability score varied from 7 to 8.04 between the different bars; the highest scores were very close, by the way, recorded in the case of 3C and 2C snack bars, both fortified with 1% FDEBP, 8.04 respectively 8. At the opposite pole is bar 1A with an average degree of acceptance of 7. Other studies that followed the degree of acceptability of some bars fortified with polyphenols from apple, pomegranate, and banana peels, moringa leaves, or tamarind seeds, observed a degree of acceptability between 6.3 and 8.5 [63,64,65]. Sensory characteristics independently influence the general organoleptic quality of food, and are generally not interdependent [64].

The degree of acceptability of a product is primarily influenced by colour, an aspect that can be easily modified by choosing the ingredients and the quantities used [66]. The colour score varied from 6.74 to 8.19 between the different bars, the lowest recorded in the control snack bars and the highest for the 3C snack bar (1% FDEBP). The least appreciated visually was 1A with a score of 6.74. In other words, the evaluators significantly preferred the darker bars. Further studies also revealed that a higher colour saturation is synonymous with consumers’ perception of being “more alive” [67].

The best taste score was evaluated in the case of the second formulation, with scores up to 1 point higher than in the case of formulations 1 or 3. The results highlight statistically significant differences, *p* < 0.001, especially for bars 2A, 2B, and 2C. It is interesting that, if in the first formulation, the addition of FDEBP did not bring significant changes in the taste evaluation, in the case of the second formulation, once the concentration of FDEBP increased, the score decreased, while for the third formulation, the addition of FDEBP increased his level of acceptability.

The panellists also prefer the second formulation regarding smell and texture, with outstanding acceptance scores for 2B. The version improved with FDEBP in the 0.5% concentration (7.99 for smell and 8.09 for texture, respectively). The lowest evaluation regarding the smell was registered in the case of bar 1A (7.38). The least appreciated texture by the participants also belonged to bar 1A (7.28).

Regarding sweetness, dates are an excellent choice in formulating snack bars. The second formulation was noticeably different from the first and third formulations. The lowest score on this characteristic was 6.84 (3A), and the highest was 8.05 (2A). The mean scores of sensory attributes between snack bar formulations are shown in Table 3.

The presented data highlight good evaluation scores for all nine variants, with statistically significant differences in all five sensory aspects: colour, smell, texture, flavour, and sweetness.

Starting from these results and considering the scientific evidence that evaluated the benefits and safety of black EB, we can say that our functional snack bar not only provides health benefits, having the potential to modulate the intestinal microbiota, but also shows a high degree of acceptance among consumers.

As for limitations of the study, the modest sample size of 81 participants, the limited diversity in demographics, and the short time emphasis can be mentioned. Employing a more extensive and diverse sample could enhance the precision of gauging consumer preferences and viewpoints. The study’s participant pool did not encompass the full spectrum of the population, displaying constraints in terms of age, gender, and geographical distribution.

## 4. Conclusions

The current study focused on the formulation of snacks enriched with EB powder, which can be a delicious way to access essential nutrients and bioactive compounds. It is a snack with prebiotic and cardiometabolic protection potential in a minimally processed form, without the addition of sweeteners or additives, and is friendly to the intestinal microbiota. 

The nutritional assessment demonstrated that the bars formulated in our research provided a complete mix of macronutrients required in a daily diet, protein (between 13.1 g/100 g and 13.8 g/100 g), carbohydrate (35.7 g/100 g—48.6 g/100 g), and fat (17.6 g/100 g—29.8 g/100 g). Fibre content ranged between 5.4 g/100 g and 6.9 g/100 g.

Based on the rheological analyses, adding 0.5% and 1% FDEBP significantly improved the structural properties of enriched functional bars, increasing their structural strength, as indicated by higher moduli, and enhanced mechanical rigidity compared to control. 

The sensory evaluation revealed positive feedback from the participants, with the best overall acceptability scores (8.04 and 8, respectively) recorded in the case of the variants with 1% FDEBP.

These findings contribute to the development of innovative and nutritious snack options that cater to consumer preferences while capitalizing on the health benefits of EB.

As for further perspectives, the impact of long-term health effects in consuming these bars is proposed, together with microbiological analysis and the proper storage conditions of the snack bars. The stability of bioactive compounds and shelf-life considerations are also under consideration.

## Figures and Tables

**Table 1:** Snack bar formulations.

Ingredients (%)	Snack Bar Symbol								
	1A	1B	1C	2A	2B	2C	3A	3B	3C
Millet flakes				13%	12.9%	12.9%	20%	19.9%	19.8%
Oat flakes	20%	19.9%	19.8%						
Walnuts	33%	32.8%	32.7%				33%	32.8%	32.7%
Peanut butter				22%	21.9%	21.8%			
Shelled hemp seeds	14%	13.9%	13.9%	13%	12.9%	12.9%	14%	13.9%	13.9%
Plum magiun	33%	32.8%	32.7%				33%	32.8%	32.7%
Dates				44%	43.8%	43.6%			
Lemon juice				8%	8%	7.9%			
FDEBP		0.5%	1%		0.5%	1%		0.5%	1%
Sum	100%	100%	100%	100%	100%	100%	100%	100%	100%

SB—snack bar; FDEBP—freeze-dried elderberry powder.

**Table 2:** Nutritional value of snack bars without FDEBP (per 100 g product).

	Protein (g)	Carbohydrates (g)	Fats (g)	Fibres (g)	Energy (Kcal)
Formulation 1	13.2	35.7	29.8	6.9	462.7
Formulation 2	13.8	48.6	17.6	5.6	400.2
Formulation 3	13.1	40	29	5.4	459.1

**Table 3:** Sensorial characterisation of snack bars (n = 81) with and without FDEBP.

Snack Bars	Acceptance	Colour	Smell	Texture	Flavour	Sweetness
1A	7.00 ± 1.11 ^a^	6.74 ± 1.13 ^c^	7.38 ± 1.39 ^c^	7.28 ± 1.26 ^c^	7.21 ± 1.57 ^b^	6.85 ± 1.53 ^c^
1B	7.40 ± 0.96 ^a^	7.51 ± 1.04 ^b^	7.44 ± 1.29 ^c^	7.52 ± 1.14 ^b^	7.20 ± 1.73 ^b^	6.96 ± 1.69 ^c^
1C	7.70± 1.02 ^b^	7.93 ± 0.98 ^a^	7.51 ± 1.38 ^b^	7.44 ± 1.19 ^b^	7.23 ± 1.66 ^b^	7.11 ± 1.59 ^b^
2A	7.95 ± 0.99 ^b^	7.89 ± 1.08 ^a^	8.17 ± 0.98 ^a^	8.25 ± 0.92 ^a^	8.25 ± 0.92 ^a^	8.05 ± 1.15 ^a^
2B	7.91 ± 1.00 ^b^	7.83 ± 0.92 ^a^	7.99 ± 1.07 ^a^	8.09 ± 0.96 ^a^	8.09 ± 0.96 ^a^	7.99 ± 1.15 ^a^
2C	8.00 ± 0.99 ^b,c^	8.12 ± 0.90 ^a^	7.98 ± 1.13 ^a^	7.89 ± 1.21 ^a^	8.01 ± 1.09 ^a^	7.98 ± 1.20 ^a^
3A	7.17 ± 1.36 ^a^	7.27 ± 1.18 ^b^	7.41 ± 1.06 ^c^	7.38 ± 0.99 ^c^	7.06 ± 1.42 ^c^	6.84 ± 1.44 ^c^
3B	7.85 ± 0.99 ^b^	7.80 ± 0.99 ^a^	7.64 ± 1.19 ^b^	7.83 ± 0.97 ^a^	7.11 ± 1.61 ^c^	7.07 ± 1.68 ^b^
3C	8.04 ± 0.93 ^c^	8.19 ± 0.88 ^a^	7.63 ±1.30 ^b^	7.60 ± 1.14 ^b^	7.26 ± 1.60 ^b^	7.17 ± 1.65 ^b^

The outcomes (presented as mean values ± SD, n = 81) exhibit distinct letters (a–c) in each column, denoting significant variations (*p* < 0.05) among the employed substrate types (as specified in Table 1), established using a one-way ANOVA, multiple comparisons test, and Tukey’s multiple range test (*p* = 0.05).

## Data Availability

Data are contained within the article.

## Supplementary Materials

The following supporting information can be downloaded at https://www.mdpi.com/article/10.3390/foods12193544/s1. Table S1: Loss tangent (tanδ) of oat based snack bars (where 1A—Oat, 1B—Oat + 0.5%EB, 1C—Oat + 1%EB) at 25 °C. Table S2. Loss tangent (tanδ) of oat based snack bars (where 1A—Oat, 1B—Oat + 0.5%EB, 1C—Oat + 1%EB) at 35 °C. Table S3. Loss tangent (tanδ) of oat based snack bars (where 2A—Millet, 2B—Millet + 0.5%EB, 2C—Millet + 1%EB) at 25 °C. Table S4. Loss tangent (tanδ) of oat based snack bars (where 2A—Millet, 2B—Millet + 0.5%EB, 2C—Millet + 1%EB) at 35 °C. Table S5. Loss tangent (tanδ) of oat based snack bars (where 3A—Millet2, 3B—Millet2 + 0.5%EB, 3C—Millet2 + 1%EB) at 25 °C. Table S6. Loss tangent (tanδ) of oat based snack bars (where 3A—Millet2, 3B—Millet2 + 0.5%EB, 3C—Millet2 + 1%EB) at 35 °C
