# Supplementary material for: Enhanced Elderberry Snack Bars: A Sensory, Nutritional, and Rheological Evaluation
Source: Foods. 2023 Sep 23;12(19):3544. doi: 10.3390/foods12193544 (PMC10572914; doi:10.3390/foods12193544)
Supplement: Supplementary file 1 [file foods-12-03544-s001.zip › foods-2621108-supplementary.pdf]

Supplementary table S1. Loss tangent (tanδ) of oat based snack bars (where 1A - Oat, 1B - Oat + 0.5%EB, 1C - Oat + 1%EB) at 25 ºC

	1A	1B	1C
**0.628**	0.369992	0.000050	-
**0.769**	0.311900	0.277241	0.344294
**0.943**	0.289936	0.271380	0.329061
**1.16**	0.275788	0.268191	0.318929
**1.42**	0.266382	0.263400	0.312137
**1.73**	0.257241	0.259853	0.306135
**2.13**	0.251087	0.259423	0.302588
**2.6**	0.245736	0.258176	0.297718
**3.19**	0.240923	0.258455	0.295372
**3.91**	0.239245	0.256433	0.295014
**4.79**	0.233766	0.255834	0.293172
**5.87**	0.232781	0.256746	0.293043
**7.19**	0.229810	0.256271	0.292549
**8.81**	0.230098	0.256520	0.293396
**10.8**	0.229398	0.257106	0.293243
**13.2**	0.226479	0.258059	0.293845
**16.2**	0.227431	0.258944	0.294912
**19.9**	0.225794	0.259608	0.295670
**24.3**	0.226016	0.260120	0.296532
**29.8**	0.225618	0.261159	0.298159
**36.5**	0.225416	0.262907	0.300761
**44.8**	0.224881	0.263754	0.301447
**54.8**	0.225699	0.264664	0.303464
**67.2**	0.225414	0.266500	0.304905
**82.3**	0.226336	0.267824	0.306052
**101**	0.226481	0.269278	0.308133
**124**	0.227081	0.270463	0.309520
**151**	0.227789	0.272357	0.311978
**186**	0.229638	0.274703	0.314390
**227**	0.231961	0.279479	0.318068
**279**	0.231993	0.279906	0.319585
**341**	0.234717	0.282070	0.322363
**418**	0.240128	0.296016	0.330771
**513**	0.292759	0.315761	0.360697
**628**	0.281519	0.328160	0.365700

Supplementary table S2. Loss tangent (tanδ) of oat based snack bars (where 1A - Oat, 1B - Oat + 0.5%EB, 1C - Oat + 1%EB) at 35 ºC

	1A	1B	1C
**0.628**	0.000050	0.121751	0.773524
**0.769**	0.301006	0.308498	0.329084
**0.943**	0.285386	0.297369	0.316423
**1.16**	0.271978	0.288730	0.306522
**1.42**	0.263583	0.281892	0.299651
**1.73**	0.256782	0.276552	0.294783
**2.13**	0.251505	0.271817	0.289971
**2.6**	0.245608	0.269571	0.286095
**3.19**	0.242021	0.267402	0.286588
**3.91**	0.239100	0.265584	0.284380
**4.79**	0.237104	0.265019	0.281655
**5.87**	0.234857	0.264374	0.282228
**7.19**	0.233042	0.264413	0.283662
**8.81**	0.231595	0.265875	0.283166
**10.8**	0.230053	0.266345	0.283843
**13.2**	0.229599	0.266902	0.286286
**16.2**	0.229111	0.268308	0.287290
**19.9**	0.227638	0.269134	0.287945
**24.3**	0.228421	0.270378	0.290578
**29.8**	0.228351	0.272435	0.291593
**36.5**	0.228824	0.273858	0.293305
**44.8**	0.228761	0.275645	0.295619
**54.8**	0.229040	0.277519	0.298380
**67.2**	0.229771	0.279708	0.300408
**82.3**	0.230596	0.282004	0.302368
**101**	0.231158	0.284023	0.304618
**124**	0.232007	0.286447	0.306936
**151**	0.232960	0.289719	0.309909
**186**	0.234566	0.292200	0.313261
**227**	0.238012	0.295743	0.315869
**279**	0.239167	0.298726	0.319274
**341**	0.239524	0.302415	0.322967
**418**	0.246081	0.307739	0.340917
**513**	0.280586	0.350125	0.348082
**628**	0.283036	0.338007	0.347765

Supplementary table S3. Loss tangent (tanδ) of oat based snack bars (where 2A - Millet, 2B - Millet + 0.5%EB, 2C - Millet + 1%EB) at 25 ºC

	1A	1B	1C
**0.628**	0.136604	0.590531	0.000050
**0.769**	0.214720	0.295702	0.323275
**0.943**	0.206485	0.297701	0.311898
**1.16**	0.200903	0.288798	0.303493
**1.42**	0.198136	0.284738	0.299184
**1.73**	0.197724	0.280554	0.295336
**2.13**	0.193040	0.277681	0.292534
**2.6**	0.191554	0.277389	0.289415
**3.19**	0.188790	0.274773	0.287177
**3.91**	0.187550	0.272242	0.288671
**4.79**	0.186700	0.273533	0.285456
**5.87**	0.185553	0.272216	0.282990
**7.19**	0.184317	0.272780	0.283674
**8.81**	0.184223	0.272096	0.283099
**10.8**	0.183563	0.272928	0.280809
**13.2**	0.183670	0.273723	0.282414
**16.2**	0.183298	0.273186	0.279888
**19.9**	0.181603	0.275354	0.280456
**24.3**	0.182829	0.274326	0.280743
**29.8**	0.180531	0.274983	0.280461
**36.5**	0.179489	0.275341	0.279903
**44.8**	0.179696	0.275567	0.279538
**54.8**	0.178263	0.276134	0.279383
**67.2**	0.177952	0.276081	0.279275
**82.3**	0.176727	0.277069	0.279475
**101**	0.176585	0.276705	0.278243
**124**	0.176649	0.277610	0.277470
**151**	0.175643	0.278300	0.276722
**186**	0.176772	0.280936	0.280044
**227**	0.179528	0.288595	0.287116
**279**	0.179637	0.286928	0.283626
**341**	0.179779	0.289801	0.283506
**418**	0.195661	0.361554	0.415994
**513**	0.243346	0.332902	0.309711
**628**	0.239749	0.345305	0.333309

Supplementary table S4. Loss tangent (tanδ) of oat based snack bars (where 2A - Millet, 2B - Millet + 0.5%EB, 2C - Millet + 1%EB) at 35 ºC

	1A	1B	1C
**0.628**	0.000050	0.000050	0.000050
**0.769**	0.249011	0.295784	0.362303
**0.943**	0.239809	0.282911	0.370865
**1.16**	0.229478	0.264064	0.330042
**1.42**	0.222085	0.253949	0.313248
**1.73**	0.217831	0.248641	0.304978
**2.13**	0.214171	0.244119	0.298445
**2.6**	0.210436	0.241071	0.295759
**3.19**	0.208382	0.238695	0.292388
**3.91**	0.206587	0.236844	0.289975
**4.79**	0.203777	0.234501	0.287803
**5.87**	0.203982	0.234699	0.287426
**7.19**	0.201954	0.232660	0.284923
**8.81**	0.201812	0.233448	0.286873
**10.8**	0.201256	0.232494	0.284666
**13.2**	0.200743	0.232044	0.283831
**16.2**	0.200512	0.232987	0.286266
**19.9**	0.201371	0.233882	0.286595
**24.3**	0.200307	0.233613	0.287152
**29.8**	0.198639	0.231978	0.285015
**36.5**	0.199855	0.233211	0.285829
**44.8**	0.199251	0.232939	0.285560
**54.8**	0.199022	0.233250	0.286136
**67.2**	0.199194	0.233022	0.284728
**82.3**	0.199572	0.233792	0.285533
**101**	0.198750	0.233081	0.284499
**124**	0.199443	0.233803	0.284709
**151**	0.199630	0.233838	0.283939
**186**	0.202425	0.236077	0.284876
**227**	0.205464	0.239394	0.288036
**279**	0.205280	0.238942	0.286707
**341**	0.206624	0.240359	0.287864
**418**	0.224836	0.298909	0.407318
**513**	0.256764	0.278115	0.308113
**628**	0.264546	0.288030	0.320014

Supplementary table S5. Loss tangent (tanδ) of oat based snack bars (where 2A – Millet2, 2B – Millet2 + 0.5%EB, 2C – Millet2 + 1%EB) at 25 ºC

	1A	1B	1C
**0.628**	0.287728	0.546190	0.000050
**0.769**	0.306522	0.242640	0.260639
**0.943**	0.279904	0.236919	0.252644
**1.16**	0.262976	0.230990	0.244440
**1.42**	0.249940	0.225702	0.239526
**1.73**	0.239580	0.222164	0.235237
**2.13**	0.231976	0.219647	0.231932
**2.6**	0.224968	0.216828	0.229164
**3.19**	0.219475	0.213975	0.226726
**3.91**	0.215511	0.213704	0.223777
**4.79**	0.211858	0.211443	0.221829
**5.87**	0.209054	0.210995	0.221136
**7.19**	0.205916	0.209480	0.219620
**8.81**	0.202338	0.208810	0.219937
**10.8**	0.202111	0.208120	0.218044
**13.2**	0.200200	0.207630	0.216917
**16.2**	0.197739	0.207018	0.216050
**19.9**	0.196023	0.207372	0.216803
**24.3**	0.194874	0.206485	0.215395
**29.8**	0.193034	0.206323	0.214711
**36.5**	0.192497	0.205135	0.214525
**44.8**	0.191835	0.204512	0.214406
**54.8**	0.189990	0.204666	0.213865
**67.2**	0.189869	0.204119	0.213370
**82.3**	0.189168	0.204260	0.213582
**101**	0.188066	0.203681	0.212883
**124**	0.187912	0.203773	0.212906
**151**	0.187781	0.204188	0.213228
**186**	0.188554	0.204825	0.215021
**227**	0.189043	0.206900	0.220474
**279**	0.189402	0.206732	0.219801
**341**	0.189881	0.207597	0.223033
**418**	0.192722	0.213498	0.305462
**513**	0.294569	0.272814	0.246574
**628**	0.217552	0.239137	0.251091

Supplementary table S6. Loss tangent (tanδ) of oat based snack bars (where 2A – Millet2, 2B – Millet2 + 0.5%EB, 2C – Millet2 + 1%EB) at 35 °C

	1A	1B	1C
**0.628**	0.846844	-	0.000050
**0.769**	0.229164	0.286749	0.263546
**0.943**	0.229086	0.275138	0.255669
**1.16**	0.225951	0.262885	0.247965
**1.42**	0.223376	0.254595	0.241883
**1.73**	0.222127	0.248657	0.235130
**2.13**	0.221092	0.243180	0.231530
**2.6**	0.220385	0.238381	0.229811
**3.19**	0.220332	0.233636	0.226566
**3.91**	0.220605	0.232447	0.227913
**4.79**	0.220516	0.229617	0.222817
**5.87**	0.220842	0.228548	0.221261
**7.19**	0.221320	0.225628	0.220275
**8.81**	0.221637	0.226422	0.221466
**10.8**	0.222894	0.224320	0.219250
**13.2**	0.223848	0.223729	0.217215
**16.2**	0.224663	0.222998	0.219425
**19.9**	0.225733	0.223010	0.219018
**24.3**	0.226049	0.222620	0.219483
**29.8**	0.227537	0.223079	0.218217
**36.5**	0.228970	0.223507	0.219218
**44.8**	0.230193	0.222710	0.220146
**54.8**	0.231729	0.223861	0.220156
**67.2**	0.232907	0.223863	0.219842
**82.3**	0.234696	0.223872	0.220624
**101**	0.235650	0.224611	0.220860
**124**	0.237447	0.225200	0.222196
**151**	0.239510	0.225487	0.224594
**186**	0.241981	0.228031	0.313494
**227**	0.246095	0.231165	0.238808
**279**	0.247129	0.232364	0.249816
**341**	0.251014	0.232941	0.298383
**418**	0.262905	0.253448	0.274045
**513**	0.309206	0.265613	0.245087
**628**	0.293829	0.261245	0.253053
